# Surprised at All the Entropy: Hippocampal, Caudate and Midbrain Contributions to Learning from Prediction Errors

**DOI:** 10.1371/journal.pone.0036445

**Published:** 2012-05-03

**Authors:** Anne-Marike Schiffer, Christiane Ahlheim, Moritz F. Wurm, Ricarda I. Schubotz

**Affiliations:** 1 Department of Experimental Psychology, University of Oxford, Oxford, United Kingdom; 2 Motor Cognition Group, Max Planck Institute for Neurological Research, Cologne, Germany; 3 Institut für Psychologie, Westfälische Wilhelms-Universität Münster, Münster, Germany; 4 Center for Mind/Brain Sciences, University of Trento, Mattarello, Italy; Max Planck Institute for Human Cognitive and Brain Sciences, Germany

## Abstract

Influential concepts in neuroscientific research cast the brain a predictive machine that revises its predictions when they are violated by sensory input. This relates to the predictive coding account of perception, but also to learning. Learning from prediction errors has been suggested for take place in the hippocampal memory system as well as in the basal ganglia. The present fMRI study used an action-observation paradigm to investigate the contributions of the hippocampus, caudate nucleus and midbrain dopaminergic system to different types of learning: learning in the absence of prediction errors, learning from prediction errors, and responding to the accumulation of prediction errors in unpredictable stimulus configurations. We conducted analyses of the regions of interests' BOLD response towards these different types of learning, implementing a bootstrapping procedure to correct for false positives. We found both, caudate nucleus and the hippocampus to be activated by perceptual prediction errors. The hippocampal responses seemed to relate to the associative mismatch between a stored representation and current sensory input. Moreover, its response was significantly influenced by the average information, or Shannon entropy of the stimulus material. In accordance with earlier results, the habenula was activated by perceptual prediction errors. Lastly, we found that the substantia nigra was activated by the novelty of sensory input. In sum, we established that the midbrain dopaminergic system, the hippocampus, and the caudate nucleus were to different degrees significantly involved in the three different types of learning: acquisition of new information, learning from prediction errors and responding to unpredictable stimulus developments. We relate learning from perceptual prediction errors to the concept of predictive coding and related information theoretic accounts.

## Introduction

The notion of the brain as a predictive machine pervades contemporary neuroscientific concepts [1]–[6]. One great achievement of the approach is that it brings perception and learning into proximity [7]. If the brain constantly predicts its sensory input [8]–[9], it has to learn correct models of its environment to achieve functional predictions [10]. This idea poses a powerful account of cortical responses [11], especially in primary sensory cortices [9] and the cortical motor network [12]. The contributions of subcortical and allocortical components, however, may not have received its due attention. The present study investigated how the caudate nucleus and hippocampus may contribute to learning in a predictive framework.

The update mechanisms of predictions are described in the predictive coding account of perception [2], [10], [13], [14]. This account recasts the brain as a Bayesian inference machine [15]. Perception relies on probabilistic models at each level of cortical hierarchy [8], [9], [11], [16]. Each of these models predicts the probability of sensory activity at the level below [8], [11], [13], [14], for the most likely states of the environment. The model sends these predictions of probable lower level activity via backward projections to the level below [2], [11]. If the sensory input at this lower level matches the predictions, the signal is filtered [9], [11], [13]. If the sensory input does not match the predictions, the difference is signaled via forward connections to the next higher level [11]. This difference is called the prediction error [8], [11]. It could also be described as the surprise at the sensory input [8], [17]–[19], linking the concept of predictive coding to information theoretic quantities. The prediction errors cause an adjustment of the model at the higher level. This adjustment pertains to learning, if the probabilities encompassed in the model and thus its predictions are altered as a result of the prediction errors [8], or if the internal model is replaced by a model that delivers more functional predictions [20]. Perceptual inference can thus lead to learning [8], [10]. What type of learning occurs depends on the reliability of information. If prediction errors accumulate, the environment is said to contain a lot of entropy [8], [18], [19]. In psychological terms, entropy can be translated to uncertainty [21]. Volatility, another measure of uncertainty, has been shown to influence learning rate [22].

Neuroscientific research on learning has discussed the interplay and competition of two learning systems [23]–[27]. One of these systems relies on the striatum, while the other is understood to be hippocampus-based. Both systems have been associated with learning from violated predictions [28]–[31]. Moreover, both systems receive projections from the midbrain dopaminergic system which seems to be involved in both systems' respective learning mechanisms [28], [31]–[33].

The hippocampal memory system is understood to be an associative mismatch detector [29]–[34], which responds when the predictions of stored representations are violated by events that were previously not associated with the stored representation [24], [35]. In clear terms this means that the hippocampus is activated more by events that relate to a known representation but differ in some regard from what has been learnt (associative mismatch) than by completely novel events [29]. The hippocampus and its underlying dopaminergic projections have been proposed to enable sequential learning [33], [35]–[37] and to code for violations of sequences [29], [38]. Lastly, new results have suggested that hippocampal activity increase is not dependent on novelty or violated predictions per se, but to uncertainty [18]. This would mean that hippocampal activity signifies the learning that oddballs can occur.

The striatum and its underlying dopaminergic projections have famously been associated with prediction errors in the context of reward-related learning [30], [31], a finding that has been replicated in humans [39], [40]. Moreover, recent imaging studies suggest that perceptual prediction errors, i.e. violated expectations unrelated to reward, also activate the striatum [41]–[43].

**Figure 1:**
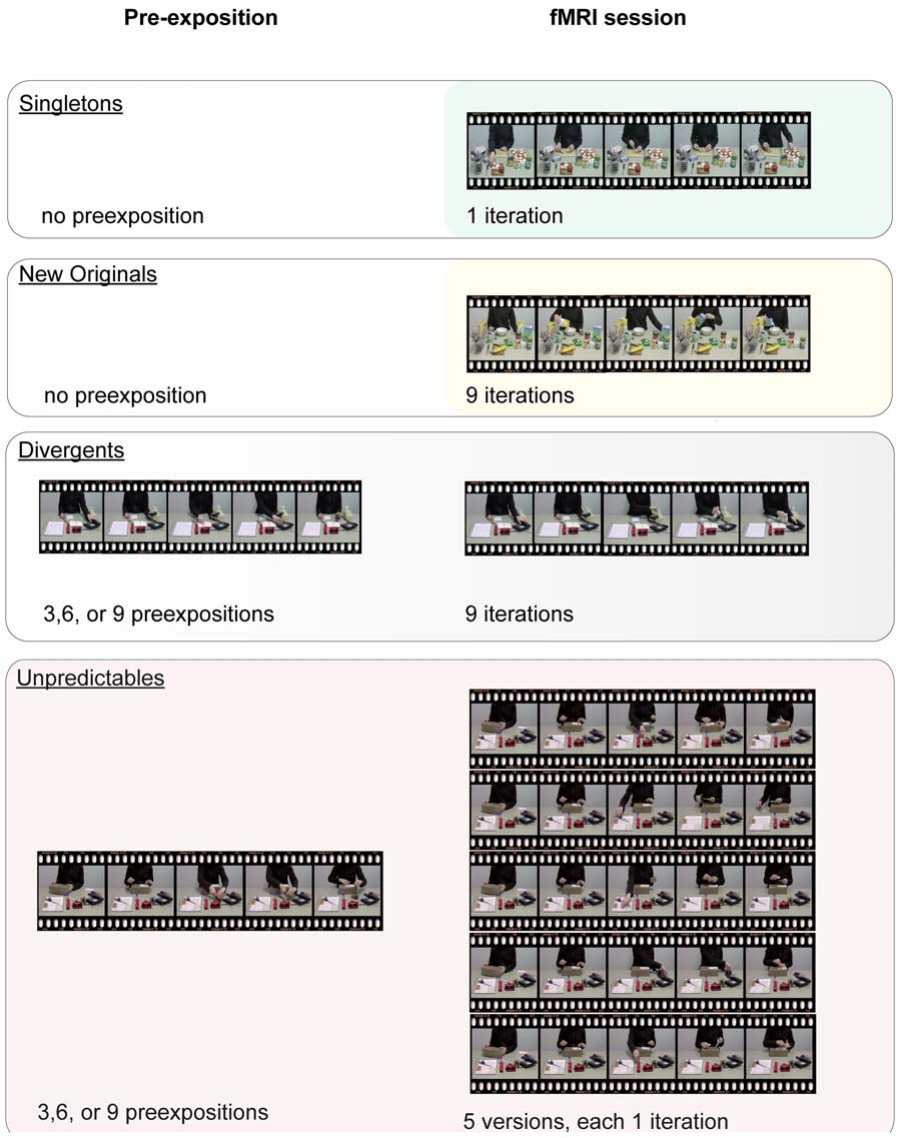
Examples for the experimental conditions of interest (*Singletons*, *New Originals*, *Divergents*, and *Unpredictables*), and their respective number of preexpositions and iterations during the fMRI. Left hand side: pre-exposition; right hand side: fMRI session. An additional category were originals (not displayed) that were shown 3, 6, or 9 times previously to the fMRI and in the identical version 9 times during the fMRI.

The current study aimed to dissociate the contributions of the hippocampal and striatal systems to different types of learning that are marked to different degrees by novelty and prediction errors. We presented subjects with videos of everyday actions that were either entirely new to the participant, were related to a known movie but then repeatedly shown in a different version, or were related to a known movie and but then repeatedly shown in different versions (Figure 1). The first type of learning that was investigated was the acquisition of new representations (we will call these representations internal models). The internal models encompassed actions and were learned through repeated exposition of the action movies (*new originals*, hereafter). The according activity change basically pertains to the adaptation of novelty responses, signified by an attenuation of the BOLD response. The second type of learning we investigated was the adaptation of predictions when the expectations of a model were violated by a divergent version of the action (*divergents*, hereafter), which was thereafter repeated. Lastly, we investigated the response to constant violation of a model by unpredictable versions of the according action movie (*unpredictables*, hereafter). This last manipulation did not allow predicting the content of a model, corresponding to a type of learning that is signified by a lot of uncertainty.

We hypothesized that the hippocampal memory system should be activated to a larger extend by associative novelty than novelty per se and thus show more activity towards the unpredictable movies and divergent movies than the novel movies. Moreover, in line with the results reported by Strange and colleagues [18] we expected the hippocampus to be responsive to the entropy that resulted from repeated violations [18].

With regard to the striatal responses during learning, we focused on a subdivision of the caudate nucleus that was previously associated with perceptual prediction errors [41] and expected this part of the striatum to be responsive to prediction errors but not to respond to novelty. We therefore predicted that activity in this caudate nucleus subdivision should decrease during repeated presentation of the same divergent model. We also predicted that this area should be activated more by the unpredictable movies that entail an accumulation of prediction errors than by the divergent movies. Lastly, with regard to the midbrain dopaminergic system, we predicted firstly, that the habenula would mirror the caudate response. This prediction is derived from our own data that has shown that the habenula mirrored caudate responses towards prediction errors [41], a finding that extends the classical view that this area is only activated towards prediction errors of a negative valence. Secondly, we investigated exploratively whether the substantia nigra, the dopaminergic input region to caudate and hippocampus, would yield activity in line with one or both structures, or would show a separate response pattern.

## Materials and Methods

### 2.1 Subjects

19 right-handed, healthy participants (7 women, age 22–30 years; mean age 25.3 years) took part in the study. The participants were right handed as assessed with the Edinburgh Handedness Inventory [44]. The experiment was approved by the local ethics committee of the University of Cologne and was in accordance with the Declaration of Helsinki. All participants were health screened by a physician and gave written informed consent.

### 2.2 Stimuli and Task

The stimulus material contained 37 different movies of 8 to 12 seconds length (mean 9.2 sec; standard deviation 1.39 sec). The movies were shot from the third-person perspective, not showing the actor's face. They contained every-day actions taking place at a table. Most movie scripts, e.g. making a sandwich, existed in 2 versions (*divergents*). Some movie scripts existed in 6 different versions (*unpredictables*). All of these scripts had an identical beginning, but started to diverge at some individual point, whereafter no commonality existed (Figure 1). Each movie script was filmed so many times, that the exact same shot of each script occurred only once during the pre-experimental and the experimental session. This method was employed to minimize surface-similarities between the movies and avoid surface-reference perceptual priming.

The experiment consisted of a pre-experimental exposition of most action movies and an fMRI session starting 15 minutes after the end of the pre-exposition. During the pre-experimental exposition session, participants were seated in a sound-attenuated chamber facing a computer screen. Distance to the screen was adjusted to ensure that the video displayed on the screen was larger than 4° of visual angle, but did not extend 5° of visual angle (depending on whether the participants moved their heads slightly). The participants watched 27 scripts, a third of which was displayed three times, another third six times and the last third nine times, in a randomized fashion over the course of the 28 minutes lasting session. As mentioned above, the participants watched one version of each script; but each repetition was another shot of the same script (minimal distance between two repetitions, or shots, of one script was 4 different scripts). Questions concerning whether a specific action had occurred in the immediately preceding script (e.g. “grasping an apple?") were posed on average after every fifth script (minimum one movie, maximum 11 movies between questions, standard deviation 2.1 movies) to ensure ongoing attention to the stimulus material. Participants received visual feedback for 400 ms on whether they had answered correctly, incorrectly, or too late. After pre-exposition, participants were transferred directly to the fMRI chamber.

### 2.3 FMRI session

36 different scripts appeared in the fMRI session. Each script was repeated over the experiment. Nine scripts that had previously been displayed during the pre-exposition were now displayed nine times each in the same version (*originals*) as in the pre-exposition. Another nine of the pre-experimentally shown scripts were presented nine times in the fMRI session in different, but always the same different version (*divergents*). Another nine scripts appeared in five different versions during the fMRI. Each of these different versions was displayed only once (*unpredictables*). One third of all movies (including the *originals*, the *divergents* and the *unpredictables*) had previously been displayed three times each, another third six times each, and one third nine times each. The design moreover encompassed three scripts that were repeated nine times during the fMRI session and completely new to the participants at first exposure (*new originals*, hereafter). Finally, there were six single movies that were displayed only once and had not been pre-exposed previously (*singletons*, hereafter) (Figure 1; Table 1). The same type of question as in the pre-exposition appeared during the fMRI, on average following every fifth movie. Importantly, these questions did not draw attention to possible differences between the versions of the movies (they were not indicative of the fact that *unpredictables* or *divergents* existed or whether the current movie belonged to either category). Apart from the question trials, the design also encompassed null-events. Null-events consisted of the display of the grey background screen for 10 seconds. Immediately after the fMRI session, participants filled in a questionnaire encompassing a free-recall task for the movie scripts.

**Table 1:** Overview of conditions and number of expositions.

Condition	No. of scripts that appeared in the respective condition	No. of preexpositions for each script on the condition	Iterations of the script in its original or complementary versions during fMRI	Repetitions of the original (pre-fMRI version) during fMRI	Repetitions of one specific version during fMRI
*Originals*	9	3, 6, or 9	9	9	9
*Divergents*	9	3, 6, or 9	9	-	9
*Unpredictables*	9	3, 6, or 9	5	-	1
*New Originals*	3	-	9	-	9
*Singletons*	6	-	1	-	1

### 2.4 Data Acquisition

The functional imaging session took place in a 3T Siemens Magnetom Trio scanner (Siemens, Erlangen, Germany). In a separate session, prior to the functional MRI, high-resolution 3D T-1 weighted whole-brain MDEFT sequences were recorded for every participant (128 slices, field of view 256 mm, 256 by 256 pixel matrix, thickness 1 mm, spacing 0.25 mm).

The functional session engaged a single-shot gradient echo-planar imaging (EPI) sequence sensitive to blood oxygen level dependent (BOLD) contrast (28 slices, 4 mm thickness, 0.6 mm spacing; in-plane resolution of 3×3 mm) parallel to the bicommisural plane, echo time 30 ms, flip angle 90°; repetition time 2000 ms; serial recording). Following the functional session immediately, a set of T1-weighted 2D-FLASH images was acquired for each participant (28 slices, field of view 200 mm, 128 by 128 pixel matrix, thickness 4 mm, spacing 0.6 mm, in-plane resolution 3 by 3 mm).

### 2.5 FMRI Data Analysis

Functional data were offline motion-corrected using the Siemens motion protocol PACE (Siemens, Erlangen, Germany). Further processing was conducted with the LIPSIA software package [45]. Cubic-spline interpolation was used to correct for the temporal offset between the slices acquired in one scan. To remove low-frequency signal changes and baseline drifts, a high-pass filter was applied. The filter length was adapted to the rate of occurrence of the rarest event and was different for the analyses containing *new originals* compared to the other analyses. The filter in the contrasts investigating only *unpredictables* and *divergents* was set at 1/85 Hz. The (parametric) contrasts containing new originals were high-pass filtered at 1/90 Hz. The matching parameters (6 degrees of freedom: 3 rotational, 3 translational) of the T1-weighted 2D-FLASH data onto the individual 3D MDEFT reference set were used to calculate the transformation matrices for linear registration. These matrices were subsequently normalized to the standardized Talairach brain size (x = 135 mm, y = 175 mm, z = 120 mm [46]) by linear scaling. The normalized transformation matrices were then applied to the functional slices, to transform them using trilinear interpolation and align them with the 3D reference set in the stereotactic coordinate system. The generated output had thus a spatial resolution of 3 by 3 by 3 mm. A spatial Gaussian filter of 5 mm FWHM was applied.

The statistical evaluation was based on a least-square estimation using the general linear model (GLM) for serially auto-correlated observations [47]. Temporal Gaussian smoothing (4 seconds FWHM) was applied to deal with temporal autocorrelation and determine the degrees of freedom [47].

The design matrices were generated by hemodynamic modeling using a δ-function and its first derivate. The onset vectors in the design matrices were modeled in a time-locked event-related fashion and set to the point in time (hereupon ‘breach’) when the movie (in the conditions *divergents* and *unpredictables*) differed from its original pre-experimental exposition version. The originals and new originals were modeled after the point in the movie that would have been the breach, if they had been displayed in their complementary version. This pseudo post-breach modeling was employed for the *originals* and *new originals*, as all scripts were counterbalanced in their assignment to conditions across participants. Thus some participants could have encountered in the function of *divergent* what to others was the *original*, or even *new original*. We did this to ensure that the measured effects did not stem from the identity of scripts or comparative length, but solely their assigned condition in the experiment. The breach had previously been visually timed to the moment when movement trajectories revealed that either the manipulation or the reached-for-object was different from that in the *originals*. The length of the modeled events corresponded to the length of the script from the breach to the end of the script (mean: 6.57 sec; STD: 1.78 sec).

### 2.5.1. Region of interest (ROI) definition

**Figure 2:**
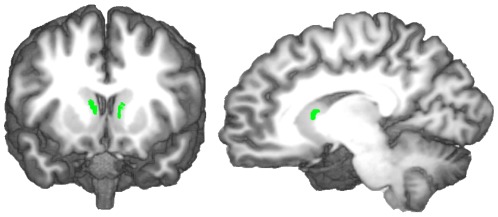
Reconstructed, color-coded caudate ROIs in a 3-D rendered brain.

**Figure 3:**
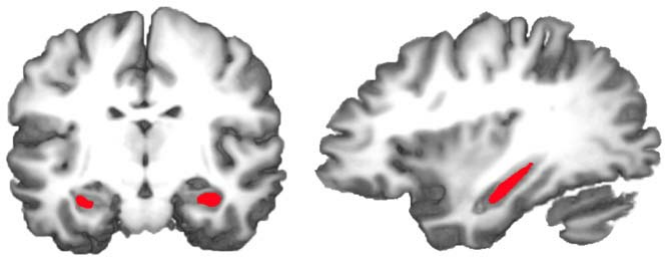
Reconstructed, color-coded hippocampal ROIs in a 3-D rendered brain.

**Figure 4:**
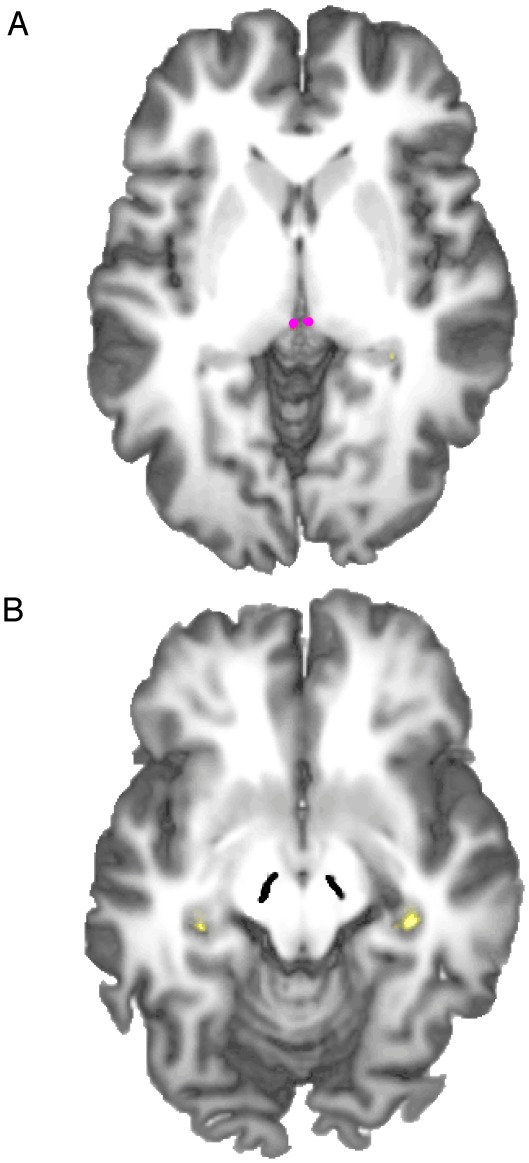
Reconstructed, color-coded habenular (A) and nigral (B) ROIs in a 3-D rendered brain.

We used the 3D T1-weighted whole-brain scans of each participant to individually segment four ROIs: left and right caudate nucleus (Figure 2), the left and right hippocampus proper (Figure 3), the left and right habenula, and the left and right substantia nigra (Figure 4). The habenula, substantia nigra and hippocampus ROIs were delimited according to anatomical landmarks. The caudate ROI was created using the coordinates of the peak voxels activated for violated predictions in a previous study [41] and choosing a radius of 4 voxels. The resulting 3-D area was then clipped in each brain individually to exclude the internal capsule and ventricles. In 3 participants, clipping the caudate ROIs to exclude the ventricles and internal capsule left nothing of the caudate ROI remaining. These participants were therefore excluded from the analysis.

The fMRI data analysis proceeded in two steps. In a first step, we modeled each condition of interest (*divergents*, *unpredictables* and *new originals*) parametrically. To that end, we generated three separate design matrices, each containing three event types, two times the movie type of interest and null-events. For example, the design matrix for *unpredictables* contained as a first event type all *unpredictables* with an amplitude vector of one. As a second event type, it contained all *unpredictables* with an amplitude vector corresponding to the specific script's iteration in the fMRI session. (The first iteration of one script was assigned an amplitude of five, the second the amplitude of four, and so forth; this regressor will hereafter be called linear parametric regressor). The last event type in the design matrix were null-events, assigned an amplitude of one. The same set up applies to the design matrices for the linear parametric attenuation modeling of *divergents* and *new originals*. In a second step, we contrasted the *unpredictables* with the *divergents* and the *divergents* with the *new originals* to investigate the relative and persistent involvement of the hippocampus proper and the striatum, i.e. caudate nucleus, in the processing of the different movie types. Thus, the fourth design matrix contained as the first event-type all *unpredictables*, each with a vector amplitude of one, as the second event-type all *divergents*, with a vector amplitude of one and lastly as a third event-type all null-events with a vector amplitude of one. The fifth design matrix contained the event-types *divergents*, *new originals* and null-events, all modeled with a vector amplitude of one. The sixth analysis contrasted 12 randomly chosen *unpredictables* (each with an amplitude vector of one) with the first presentation of the *new originals* and *singletons* (with the same amplitude) and also contained null-events.

### 2.5.2. Modeling information theoretic quantities

**Figure 5:**
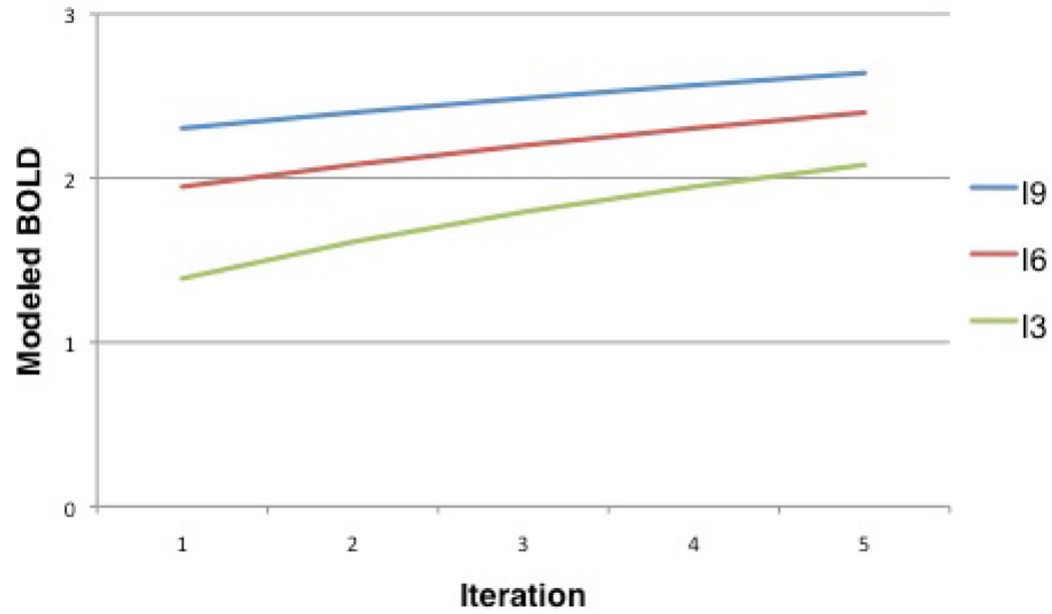
Modelled BOLD for surpise over the iterations of unpredictables in the fMRI session. I3: surprise for the 3 times pre-exposed; I6: surprise for the 6 times pre-exposed; I9: surprise for the 9 times pre-exposed unpredictables.

**Figure 6:**
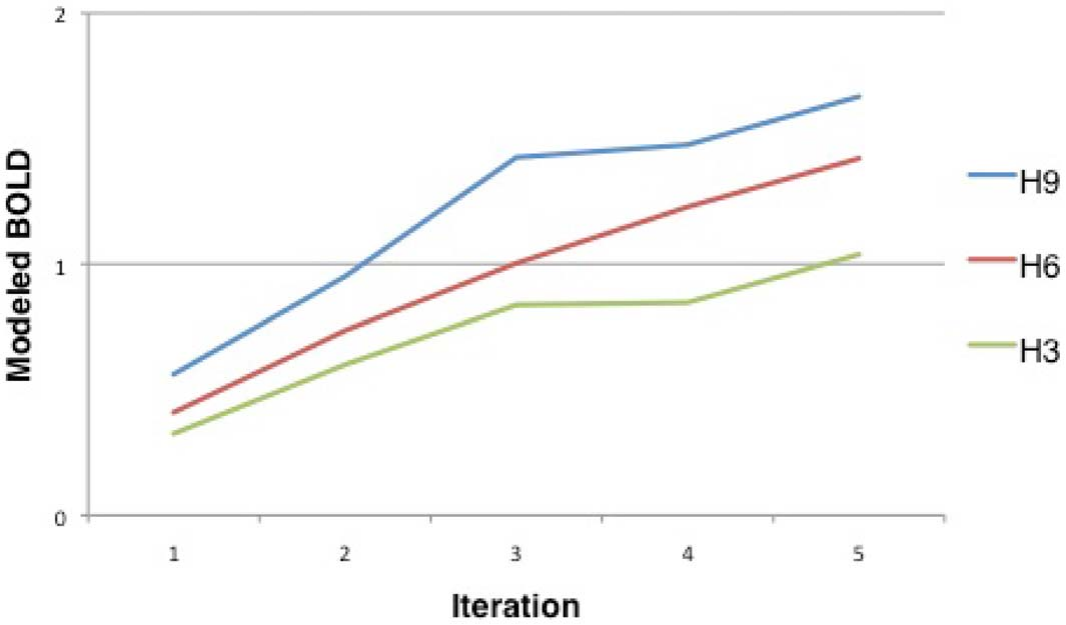
Modelled BOLD for entropy over the iterations of unpredictables in the fMRI session. H3: entropy for the 3 times pre-exposed; H6: entropy for the 6 times pre-exposed; H9: entropy for the 9 times pre-exposed unpredictables.

We calculated the responses of all four bilateral ROIS to the surprise (Figure 5) and the Shannon entropy (Figure 6) ascribed to the content-development of the *unpredictables*. We assumed that the brain should behave like an ideal observer and hence ascribe the probability of an item according to:
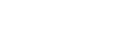
This model is in close keeping with the approach taken by Strange and coworkers [18]. The probability (“p") of the observation (“x") of a specific movie version (“i") is calculated as the number of times (“n") the script has appeared in exactly that version (“i") so far (“j") divided by the sum of appearances in all versions (“k") that have appeared so far (“j"). The addition of the value 1 shape a Dirichlet distribution, that accords to an ideal observer. The information theoretic quantities thus concern probabilities as they are defined in a Bayesian framework.

Following previous approaches [17]–[19], surprise (“I") at an outcome (“x_i_") was calculated as:
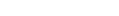
This term, also known as the ‘negative evidence’ since surprise is calculated as the negative logarithmic (“−ln") probability (“p(x_i_)") of a specific movie version. It indicates the amount of information that is conveyed by the observation [8].

Another important construct that describes the influence of observations is Shannon entropy. Shannon entropy is again a term derived from information theory [19] (but see [21]) and describes the average surprise in a series of observations [17]. Shannon entropy is therefore mathematically calculated as:
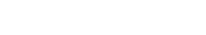

[17]–[18], [21]. Entropy (“H") at the observation (“x_i_") is thus the negative probability (“-p(x_i_)") of the observation of a movie script multiplied with its logarithmic probability (“ln p(x_i_)"), summed for all versions (“i-k") that are known to have occurred. (We employed the natural logarithm, but binary approaches have been used (cf. [18]). If all observations are equally likely and appear equally often, each event is surprising, as it cannot be predicted [17]. This is the setup of the highest Shannon entropy. If Shannon entropy is large, each event is very informative [8], [17], [19].

### 2.5.3 Second level analysis

The second level analysis employed a permutation analysis to correct for false-positives [48]. For all above-mentioned contrasts or parametric analyses, we calculated 2000 different one-sample *t* tests for each of the four ROIs. The important manipulation consisted in a different reversal of experimental and control condition in one to 16 subjects in all 2000 *t* tests. We thus conducted a permutation of beta values, yielding different t-values for each reversed assignment of the two conditions in the contrasts on the subject level, as arbitrary relabeling of events is not suggested for fMRI [48].

It can thus be determined, whether the analysis that agrees with the experimental setup in all participants reaches a higher *t*-value than randomly permuted analyses. This would then indicate, that the activity revealed in the contrast is best accounted for by the contrast between experimental and control condition and not due to noise. The benefit of such a bootstrapping approach is that the *t* tests do not assume a Gaussian distribution, but calculate the distribution based on the variance in the data [48]. This is important, as the use of a Gaussian distribution does not necessarily fit activity in a spatially circumscribed ROI. The cut-off *t* (*t_cri_*_t_) for significance testing was set at p = .05. This means that 1900 permutations of the assignment between subjects and conditions must result in a lower *t* than the original experimental assignment wherein the control condition is used as control condition and the experimental condition used as experimental condition for all 16 subjects.

### 2.5.4. Orthogonal model approach

In the analyses as laid out above, different design matrices were used for each contrast, yielding maximum power to the specific analysis. However since a number of analyses pertain to the same events *(unpredictables)*, but apply different models, we subdued the fMRI data to additional analyses. These additional analyses made use of a unified design matrix and orthogonalised the modeled effects of entropy, surprise, and the linear parameter. To that end, we generated one design-matrix as follows: The *unpredictables* appeared in 4 event types. In the baseline entry, all amplitudes were set to one. This was the first event type. For the second event type, the amplitude vector was set corresponding to the item-specific entropy. The third entry carried an amplitude vector corresponding to surprise orthogonalised to entropy. The fourth entry carried an amplitude vector according to the parameter signifiying linear decrease, in a first step orthogonalised to entropy and in a second step orthogonalised to surprise. The last entry of this design matrix were null-events. The second-level permutation-based analysis was the same as for the main contrasts (cf. 2.5.3).

### 2.6 Behavioral data analysis

After the fMRI session, participants were asked to recall as many actions as they could remember. To test if the different actions were differently well remembered depending on their condition, these free recall rates were analyzed. Therefore, it was counted how many movies of each condition were recalled by each subject, which resulted in the absolute number of recalled *divergents*, *unpredictables*, *new originals*, *originals* and *singletons*. Furthermore, it was counted how often each of the recalled movies had been seen during the experiment (during the pre-exposition and the functional scanning). The number of expositions was aggregated for each version of the movies, i.e. divergent movies have been exposed 3+9, 6+9, or 9+9 times (pre-exposition+functional scanning), whereas all new originals had been exposed 9 times (during the functional scanning). To calculate the average number of exposition of the recalled movies of one condition, the numbers of expositions of the recalled movies were summed and then divided by the number of the recalled movies. The inferential analysis was performed in three steps:

At first, the influence of the exposition frequency was partialed out by running a multiple regression with the sum of the recalled actions (per condition) as dependent and the number of pre-expositions as independent variables. The standardized residuals of this analysis, i.e., the information that was not explained by exposition frequency, served as dependent variable in the analysis of the condition effect. To that end, a repeated-measures ANOVA was calculated with the factor condition (levels: *originals, new originals, divergents, unpredictables, singletons*).

It must be borne in mind that all unpredictable versions of one movie shared common actions in the common beginning of the script. Moreover, the objects in different versions were sometimes the same as in other versions, while the manipulation of the object differed. For instance, all 6 different versions of one particular movie (the pre-exposed version as well as the five unpredictable versions during the fMRI) contained a piggy bank. Naming a script from the *unpredictables* condition was therefore not necessarily harder than naming a script from the *originals*, *new originals* or *divergents* condition.

## Results

### 1. Behavioral results

The behavioral analysis assessed how many actions were recalled depending on the contingencies of the corresponding action movie during the experiment, i.e., whether it was completely new when encountered during the fMRI session (*new originals*), whether it was displayed in a divergent version during the fMRI compared to the preexposition (*divergents*), or whether it displayed an altered version on each iteration during the fMRI (*unpredictables*). The correlation between the sum of recalled actions per condition and the exposition frequency was significant (r = .458, p<.001). The repeated measures ANOVA on the standardized residuals of the number of recalled actions and the factor condition yielded significance (F_(2.81,50.54)_ = 3.505, p = .024; Greenhouse Geisser corrected for non-sphericity). On average, the number of recalled actions in the condition *divergents* was higher than for actions in the conditions *new originals* and *unpredictables* (number of recall of divergents = 2.26; new originals = 0.47; unpredictables = 1.53). This difference was also visible in the standardized residuals, which served as dependent variable in the ANOVA (mean residual number of recalled divergents = 0.530; new originals = −0.471; divergents = 0.182).

### 2. ROI analyses

#### 2.1. Contrast relating to acquisition

There was a significant attenuation of activity with repeated exposures of the *new originals* in the hippocampus (*t* = 1.45; *t_crit 5%_* = 0.65; p<.05). The substantia nigra showed attenuation of activity in the same parametric contrast (*t* = 2.00, *t_crit 5%_* = 1.56, p<.05). There was no significant attenuation of activity in the caudate nucleus ROI.

The substantia nigra was the only structure that showed a main effect for the processing of *new originals* vs. *divergents*. Thus, it was significantly less activated by the processing of *divergents* compared to *new originals* (*t* = −1.225; *t_crit 5%_* = −1.225; p<.05).

#### 2.2. Contrasts relating to adaptation

The hippocampal ROI revealed a significant attenuation of activity with the repeated exposure of *divergents* (*t* = 0.88; *t _crit 5%_* = 0.62; p<.05).

#### 2.3. Contrasts relating to unpredictability

Processing of the unpredictables activated the hippocampal ROI significantly more than processing of *new originals* and *singletons* (*t* = 1.65, *t_crit 5%_* = 1.60, p<0.05). In the caudate ROI there was more activity for the processing of *unpredictables* at all stages than for the processing of *divergents* (*t* = 2.33; *t_crit 5%_* = 1.76, p<0.05). Likewise, the habenula (*t* = 2.58; *t_crit 5%_* = 1.79; p<0.05) and the substantia nigra (*t* = 2.45; *t_crit 5%_* = 1.56; p<0.05) were activated more by *unpredictables* than by *divergents*. There was no attenuation with the repeated exposure of *unpredictables* in any ROI at p<0.05.

A repeated measures ANOVA testing for main effects of condition (levels: *new originals, divergents, unpredictables*) on attenuation effects in the hippocampal ROI. The repeated measures ANOVA yielded a significant main effect of condition. This effect was due to significant differences between *new originals* and *unpredictables*. Since the dependent variable reflected the slope of the attenuation, these results indicate an interaction between the course of the attenuation and the condition in the hippocampus.

#### 2.4. Modeling information theoretic quantities

The BOLD changes in the hippocampal ROI varied to a statistically significant degree with the Shannon entropy of each observation of an *unpredictable* (t = 1.83; t_crit5%_ = 1.59, p<0.05). Activity in the caudate ROI for the modeling of surprise (as defined by information theory) during the observation of *unpredictables* approached significance at p = .054 (t = 2.98;t_crit 5%_ = 3.00; t_crit 10%_ = 2.73, p<0.1)

#### 2.5 Orthogonal modeling results

Modeling entropy yielded replication of the former significant result for the hippocampus ROI during the observation of *unpredictables* (t = 1.83; t_crit 5%_ = 1.50). Modeling surprise orthogonal to entropy yielded significant activity in the caudate ROI during the observations of *unpredictables* (t = 3.24; t_crit 5%_ = 2.73) exceeding the probability of the marginal effect in the former analysis. No other ROI yielded significant activity corresponding to entropy during the observation of *unpredictables*. No other ROI yielded significant activity according to surprise orthogonalised to entropy during the observation of *unpredictables*. No ROI yielded significant activity for the modeling of a linear decrease orthogonalised to entropy and surprise during the observation of *unpredictables*.

## Discussion

Hippocampus, caudate nucleus and midbrain dopaminergic system are supposed to contribute to learning and all of these systems have been associated with learning from prediction errors. But each structure's specific contribution to learning in the absence or from the presence of prediction errors deserves further investigation. Here we used fMRI to tap these structures' roles in learning of observed action episodes. The hippocampal ROI showed a decrease in activity as hypothesized during the acquisition of a new model and the adaptation of an internal model to a changed script. Thus, both adaptation processes are signified by hippocampal decrease.

Interestingly, we found significantly higher activity for the unpredictable violation of a known model than for complete novelty, in line with the associative mismatch account [29]. Lastly, the hippocampus showed an activity increase over the course of *unpredictables* that reflects the Shannon entropy, or average surprise, elicited by the prediction errors inherent in this condition (Figure 6).

In contrast to the response pattern observed for the hippocampal ROI, the caudate nucleus ROI was significantly more activated by the processing of the prediction error profuse *unpredictables* than by the processing of the eventually predictable *divergents*. The caudate ROI also showed activity corresponding to the surprise (Figure 5) entailed by the *unpredictables*.

Finally, as predicted, the habenula reflected the caudate response to the occurrence of prediction errors in the *unpredictables*. The substantia nigra displayed novelty responses, here with regard to completely novel internal models.

### Predictive Coding and the Hippocampus

The hippocampal activity accompanied the acquisition of a new model and adaptation of an old model to change: Each observation of the *new originals* and *divergents* led to a decrease in hippocampal activity.

Activity decrease is understood to be a hallmark of learning (see [49] for a recent review). Predictive or inferential accounts of brain function explain why a decrease in activity can be regarded a sign of learning [2], [9], [11], [14]. To resurrect the picture, the brain builds models of likely perceptions [11], [14], [50], [51]. Sensory input is predicted on the basis of these internal models. The model effectively filters all anticipated information and thus modulates cortical activity to represent only surprising, informative input [2], [11]. This activity, due to prediction errors, can either cause the model to loose weight in predicting the sensory input (and thus effectively being replaced by another model, cf. [20]), or induce the change of the models' predictions [10] pertaining to learning. Decrease of neural activity over repeated iterations of a model is therefore regarded as a sign of learning [51], [52]. As the model gets better, there are less prediction errors, causing less cortical activity. The fact that the model gets more precise in predicting sensory input, and therefore more and more of the signal, means it has learnt.

Predictive coding is usually regarded to deal with current, not anticipated sensory input [12], [5]. However, viewing hippocampal activity from a predictive coding perspective reveals how predictions into the near future could be mediated. Combining sensorimotor cortical responses as explained by predictive coding [2], [11] with models of hippocampal function [36], [38] explains how predictions of consecutive events can be established and matched with sensory reality. Two functions of the hippocampus relate to this account: first of all the hippocampus is regarded to store compressed representations of cortical activity [24], [52], [53]. Secondly, it has the capability for coding sequential events [52], [54]–[56], for example in spatial navigation [23], [29], [34], [57], [ but see 58] and during learning of episodes [54], [55]. These functions relate to ‘relational representation’ [55], [56], [59], [60], [ but see 61], that means a sparse coding of cortical patterns and their relation in time and space. This coding for relation is achieved by small overlaps between the sparse representations of the cortical patterns [62].

The underlying idea is that prediction of sequential events [52], [55] and spatial navigation [63] relies on the succession of cortical patterns [64], coding for the (visual) input at a given time, and the (visual) input that should come next. To predict the next pattern in the sequence, the hippocampus can use the above-mentioned minimal overlap between the cortical representations to bind current activity to the activity pattern that is to follow. The overlap between representations is strengthened by repeatedly experiencing the sequence of cortical patterns [61], [62]. Importantly, hippocampal representations can be back-projected to the cortex, which forms the putative mechanism behind retrieval and implicit learning [36]. The predictive coding account suggests that cortical patterns are diminished once they are predicted. If one cortical pattern that is part of a compressed sequential representation was elicited by unpredicted (e.g. visual) input, this would lead to a retrieval of the stored representation (cf. pattern completion, [29], [38]) that predicts the next cortical pattern in the sequence [29], [64]. If this cortical pattern occurred, it would be effectively filtered according to the predictive coding account [49]. This filtering results in less cortical activity and this smaller extent of cortical activity may in turn cause comparatively less encoding or weight change in the hippocampus, compared to a perception that does not fit the predicted input; this account explains novelty signals and especially signals reflecting the mismatch between predictions and sensory input as unfiltered prediction errors.

We could show that long stimulus sequences, i.e., actions that are new to the observer lead to a stepwise decrease in hippocampal activity. We propose that the sequence of actions in the scripts became predictable and the associated sequence of cortical patterns resulted in a filtering of the sensory input. The decrease in hippocampal activity can therefore be understood as a sign of an increasingly valid model that predicts the course of the observed action [49], [50]. It is important to note that the predictions of sensory input entailed conceptual predictions, as the different shots of each script negated surface-similarities.

The associative mismatch account of hippocampal function [29] in fact captures the same elements as predictive coding. It predicts that anticipated input will result in lower activity than unpredicted input. Moreover, Kumaran and Maguire [29] could show that unpredicted input also elicits more activity than novel input. Thus, not novelty, but the mismatch between expected and perceived sequences activated the hippocampus [29]. This finding coined the term of “associative mismatch detector" as a description of the function of hippocampus proper. The present study extends this notion in an important manner. The unpredictable courses of known movies elicited more activity than completely new movies. The finding that novel items (*singletons* and 1^st^ *new originals*) elicit less activity than *unpredictables* that relate to a previous association can also be recast in terms of predictive coding. As described previously, predictive coding rests on Bayesian inference. That is, the first of frequently paired items starts to predict the second item with a high conditional probability. If this pairing is consistent, the brain experiences little entropy and will therefore not expect any deviations. A violation of this prediction results in a higher activation than the encounter of an action movie that is not encompassed in a recently acquired internal model, as in the case of the first new originals and singletons. If no solid internal model exists so far, the input will be filtered only to the degree that is proposed by known action semantics. In comparison to the episodic internal model trained for the *unpredictables*, the internal model for the *new originals* does not ascribe a solid probability to specific episodically acquired predictions. Thus, the mismatch signal is smaller due to more lenient semantic predictions.

### Entropy in the hippocampus

The current results suggest that the hippocampal activity reflects Shannon entropy of the unpredictable courses (cf. [19]). Shannon entropy measures the average surprise within a stimulus stream [18]. In psychological terms we can therefore regard entropy as a measure of uncertainty concerning predictions. While the responsiveness of the hippocampus to Shannon entropy replicates a result by Strange and colleagues [18], it also expands our knowledge on hippocampal function substantially. The experiment by Strange and colleagues [18] dealt with learning of statistical regularities. It did however not allow learning to predict the next item. On the contrary, it was only possible to learn to predict the rate of occurrence of items [18], [65]. On the other hand, a related study by Harrison and colleagues [65] investigated the involvement of the hippocampus in learning the likelihood of a transition between two successive items. These authors found no indication of hippocampal coding for entropy [65]. In the current study the hippocampus was sensitive to the entropy caused by unpredicted sequences of actions, thus indicating that the hippocampus is sensitive to the predictability of transitions in very complex stimuli, and without a priori knowledge of all transitions or stimuli that will occur. This latter fact seems to be relevant considering that Strange and coworkers [18] have suggested that the hippocampus does not encode the stimuli that violate predictions, but the fact that these occur. The stimuli used by Strange and colleagues [18] were all a priori known. Thus there was no need to encode their existence. But these stimuli allowed acquiring an expectation of their probability, which pertains to entropy. On the contrary, the current study employed action movies and violations stemmed from previously unassociated actions within the sequence. If these actions had not been encoded, future violations and the entire unpredictability could not have been detected. In fact, if the content of violation had not been encoded at all, the responses towards the *unpredictables* would have mirrored the responses towards the *divergents*.

Having said that, it is interesting that the free recall rates for *divergents* surpassed that for *unpredictables*, suggesting a less successful encoding of the *unpredictables*. This finding may be not surprising, given the fact that *unpredictables* did not possess the reliability to enable future valid predictions. We thus find tentative evidence that while stimulus sequences exposing high Shannon entropy are encoded to a certain degree, the encoding is not as successful as that for low-entropy or stable sequences. In similar vein, an interesting study by Davis and colleagues [66] found that during a category learning task, hippocampal BOLD activity was significantly correlated to modeled entropy of the stimulus stream. Those items that were later better remembered (exception to the rule items) also significantly drive entropy in a stimulus stream.

Based on the results of the present study, we propose that the hippocampus adapts its models of sequential sensory input as implied by the associative mismatch account [29]. Moreover is the hippocampus sensitive to the uncertainty under which it receives information and encodes the uncertainty-eliciting input to a specific degree.

### The caudate nucleus in perceptual prediction errors

The caudate nucleus showed a higher response to *unpredictables* than to *divergents*. Each *unpredictable* contained a breach of expectation on the content level, that is the sequence of actions. But only the first *divergent* contained a breach of expectation on the content level while each subsequent *divergent* version of the same movie repeated the same divergence compared to the original script. On a higher level of description, each breach of expectation of the *unpredictables* that occurred after the second iteration was fully predictable as such, (albeit not predictable with regard to the post-preach content). Caudate nucleus activity was therefore driven by prediction errors on the content level, indicating a lack of meta-learning. We could not establish a linear decrease in caudate activity that would further argue in favor of a prediction error account, as it would signify a decrease in activity as predictions become more reliable. However, this null-finding could also be due to a non-linear decrease over the iterations of divergents. Caudate signaling of prediction errors is noteworthy in itself, as only few fMRI studies have discussed the striatal involvement in not reward related prediction errors [41]–[43]. The still dominant account for striatal functioning is the *temporal difference model* that is usually associated with reward related learning [3], [31]. Only one recent study has applied prediction errors in terms of predictive coding to striatal function [42]. The results of the present study substantially foster the alternative, not reward related, understanding of striatal prediction error signaling: the indication of prediction errors on a perceptual level, irrespective the presence of reward or punishment [42] and possibly associated with the amount of surprise that a prediction error entails. On a related note, it is interesting that the habenula mirrored the caudate activity. This result substantiates our previous finding [41] of the habenula's involvement in coding for perceptual prediction errors. This result and its replication are highly interesting, as it expands the generally finding that the habenula codes for punishing or “worse than expected" outcomes [67]. In close keeping with an argument put forward by Friston and colleagues [68] prediction errors can concern the valence of an outcome. However, the involvement of the habenula in perceptual prediction errors could indicate that prediction errors as an outcome of a predictive process can have a valence themselves, possibly motivating the improvement of internal models.
